# Survey on patients’ attitude towards the nutritional counselling in the dental setting

**DOI:** 10.1038/s41405-024-00229-0

**Published:** 2024-06-11

**Authors:** M. Iriti, G. Spallino, R. Franchini, M. Rigoni, P. Muti, G. Lodi, A. Sardella, E. M. Varoni

**Affiliations:** 1 Dipartimento di Scienze Biomediche, Chirurgiche ed Odontoiatriche, University of Milan, Milan, Italy; 2 Odontostomatologia, ASST Santi Paolo e Carlo – Presidio Ospedaliero San Paolo, Milan, Italy; 3 Fondazione IRCCS Ca’ Granda Ospedale Maggiore Policlinico, Milan, Italy

**Keywords:** Nutrition and diet in dentistry, Oral diseases

## Abstract

**Aim:**

A healthy diet could help to prevent both oral and systemic diseases, with dentists and nutritionists supplementing their skills. The dental setting, where patients periodically refer to seeking oral health care, represents a powerful opportunity for nutritional counselling. To the best of our knowledge, no study is available on patients’ attitudes towards dietary counselling in the dental setting. This cross-sectional study investigates patients’ attitude towards receiving nutritional support within the dental setting and it elucidates whether a transdisciplinary approach would be well accepted.

**Materials and Methods:**

A questionnaire was administered to patients attending three different clinics: a private clinic, a hospital dental clinic of the national healthcare system and the private dental practice within the same hospital.

**Results:**

Three hundred thirteen questionnaires were collected. Most dental patients acknowledged receiving nutritional advice from both dentists and nutritionists. The nutritionist within the dental setting was positively perceived, providing useful advice to prevent oral and systemic diseases and also drawing up a diet with periodic follow-ups.

**Discussion and conclusion:**

These findings support the positive attitude of patients towards receiving nutritional counselling within the dental setting. The dental clinics can be pivotal in oral and systemic disease screening and prevention and a multidisciplinary approach is highly encouraged.

## Introduction

In 2017, the World Health Organization (WHO) promoted the “One Health” approach, where the transdisciplinary way of working represents a crucial aspect of achieving optimal health outcomes. Oral health and systemic health are closely related and oral health can be considered “a key indicator” of overall health, sharing modifiable risk factors with many other non-communicable diseases, such as diabetes [1–3]. WHO also emphasized the crucial role of a healthy diet in the prevention of several chronic diseases, with a call for action on global nutritional programs [4]. Nutritional counselling, together with physical exercise, is pivotal for preventing both type 2 diabetes and periodontal diseases, besides reducing overweight, obesity, and cardiovascular risk [5].

Like many non-communicable diseases, such as diabetes, cardiovascular diseases, cancer, and osteoporosis, dental diseases are significantly influenced by dietary habits: caries can be prevented by limiting the frequency and amount of sugar intake [6], while tooth erosion by dietary acids contained in some beverages or other acidic foods further contributes to tooth destruction [7]. Along these lines, recent reviews highlighted the role of macronutrient intake (e.g., excessive carbohydrates or polyunsaturated fat intake, and deficient protein intake) and of micronutrient intake (e.g., deficiencies of vitamin C and B12, minerals, and trace elements) in the risk of periodontal diseases [8–10]. Recommendations for oral health practitioners included dietary advice about the intake of fish oils, fibre, fruit, and vegetables, and about limiting refined sugars for periodontal and systemic health preservation [9, 11–13].

The role of the dental setting in providing nutritional advice is important not only to promote oral health, but also in preventing dietary-related systemic diseases; in this perspective, oral healthcare professionals may significantly contribute to diabetes screening and health education [14], and to address overweight and obesity [15]. The American Dental Association (ADA), thus, recommends dental practitioners to stay abreast of the latest evidence-based nutrition recommendations and nutrition-related screening, counselling and referral techniques, and it encourages collaborations among dentists, dieticians, and other nutrition experts, to raise interdisciplinary awareness about the relationship between diet, nutrition, and oral health [16].

Although most oral health professionals consider it important performing a medical screening during their practice [17–19], patient’s opinions and knowledge about the importance of dietary advice when seeking for dental care have never been in-depth explored. There is currently a need to better elucidate the perception of individuals towards receiving nutritional counselling in the dental setting, in the perspective of improving the global assistance to patients and promoting general wellbeing. Therefore, the primary aim of this study was to investigate the patients’ attitude towards receiving a nutritional support within the dental setting, in order to clarify whether a transdisciplinary approach could be positively perceived. The secondary aim was to assess differences among three different dental settings, i.e. private practice, healthcare system within the hospital, and private practice within the hospital (called *intra moenia)*.

## Materials and Methods

### Study design, setting, and population

This survey was designed as a cross-sectional study involving a population of random patients, who referred to dental settings in Milan and Lecco (Italy). Patients were enrolled by their attendance to three different clinics (convenience sampling): a private clinic in Lecco (Centro Odontostomatologico, Lecco, Italy) (Group 1); a dental clinic of the national health system in Milan (ASST Santi Paolo e Carlo, Milan, Italy) (Group 2), and the private *intra-moenia* practice within the same dental clinic (Group 3). This survey was conducted from January 2023 to May 2023.

Inclusion criteria were age over 18 years old and willing to participate in the survey, while exclusion criteria were age under 18 years old or refusal to participate.

### Questionnaire used for the survey

A semi-structured questionnaire consisting of demographic data and six focused questions (Supplementary Appendix 1) was given to random patients referring to the three dental settings, after obtaining their informed consent. The questionnaires were self-administered by patients, anonymously, to maintain the privacy and confidentiality of all information collected in the study.

The items in the questionnaire were, then, checked for their suitability and adaptation by two experienced dieticians and four dentists. Due to the lack of a similar questionnaire in literature, a group of 12 patients, attending private and public dental clinics, preliminarily tested the questionnaire. All the authors achieved the consensus on the final version. The results of this preliminary process were not included in the main study.

### Body mass index (BMI) calculation

Body Mass Index (BMI) was calculated as follows:

$${{{{{\rm{BMI}}}}}}={{{{{\rm{weight}}}}}} \, ({{{{{\rm{kg}}}}}})/{{{{{\rm{height}}}}}} \, ({{{{{{\rm{m}}}}}}}^{2})$$

Cut-off values for underweight, normal weight, overweight, and obesity were set according to WHO. Values under 18.49 corresponded to underweight (in particular: <16.00 severe underweight; 16.00–16.99 visible underweight; 17.00–18.49 slightly underweight); values in the range 18.50–24.99 were considered normal weight; values between 25–29.99 overweight; values over 30 corresponded to obesity (in particular: 30.00–34.99 I class obesity, 35.00–40.00 II class obesity, >40.00 III class obesity).

### Statistical analysis

The sample size was calculated using the online tool http://www.raosoft.com/samplesize.html, considering the general adult population living in Milan and Lecco in the period when the study was conducted (data source: ISTAT). The recommended sample size was 271 (margin of error: 5%; confidence level: 90%; response distribution: 50%), distributed two-third from Milan and one-third from Lecco. Each enrolled participant was considered as a statistical unit. Descriptive statistical analysis was used to describe the items included in the survey. Means and standard deviations were used for continuous variables, and numbers and percentages were used to describe dichotomous and categorical data. A subgroup analysis (according to sex or BMI) was performed to understand potential modifiers influencing the answers, under the hypothesis that the sex or over/underweight of respondents could influence the results. Baseline characteristics among the three groups and questionnaire responses were compared using the most appropriate test (i.e., continuous variables were compared by using one-way ANOVA and categorical variables were compared by using the χ^2^ test or Fisher’s exact test).

Data were analysed using Excel^®^ (Microsoft Excel), and Stata software (StataCorp, Texas, USA) version 17.0. The *p*-value for statistical significance was set at 0.05.

## Results

Three-hundred thirteen patients (123 males and 190 females) participated in the survey (mean age: 53 ± 17 years; range: 18–87 years). In Group 1 (dental setting: private practice), questionnaires were filled by 109 patients (mean age: 51 ± 15 years; range: 18–82 years; 24 males and 85 females). In Group 2 (dental setting: healthcare system within the hospital), questionnaires were filled by 104 patients (mean age: 53 ± 18 years; range: 21–80 years; 50 males and 54 females). Group 3 (dental setting: *intra moenia* private practice within the hospital) included 100 questionnaires (mean age: 54 ± 19 years; range: 19–87 years; 51 females and 49 males).

Table 1 summarizes the main patients’ socio-demographic data at baseline, divided into three different settings.

**Table 1:** Baseline socio-demographic characteristics of study participants, divided in the three dental settings: Group 1 = private practice; Group 2 = healthcare system within the hospital; Group 3 = private practice within the hospital (*intra moenia*).

	All patients	Group 1	Group 2	Group 3	*p*-value
Age, mean (SD), years	53 (17)	51 (15)	53 (18)	54 (19)	0.56
Male, *n* (%)	123 (39)	24 (22)	50 (48)	49 (49)	<0.01
Female, *n* (%)	190 (61)	85 (78)	54 (52)	51 (51)	
Height, mean (SD), m	168 (9)	166 (9)	169 (9)	169 (10)	0.06
Weight, mean (SD), kg	69 (15)	65 (17)	71 (14)	71 (14)	<0.01
Body Mass Index-BMI, mean (SD), kg/m^2^	24 (5)	23 (7)	25 (4)	25 (4)	<0.01
Working occupation:					
Unemployed/housewife, *n* (%)	121 (44)	34 (35)	48 (59)	39 (39)	0.01
Employed, *n* (%)	146 (52)	60 (61)	32 (39)	54 (55)	
Students, *n* (%)	12 (4)	4 (4)	2 (2)	6 (6)	
Number of medications assumed daily, mean (SD)	1.3 (2.1)	0.7 (1.2)	1.8 (2.2)	1.6 (2.4)	<0.01

Group 1 = private practice; Group 2 = healthcare system within the hospital; Group 3 = *intra moenia* private practice within the hospital; *SD* Standard deviation, *m* meters, *kg* kilograms.

**Fig. 1:**
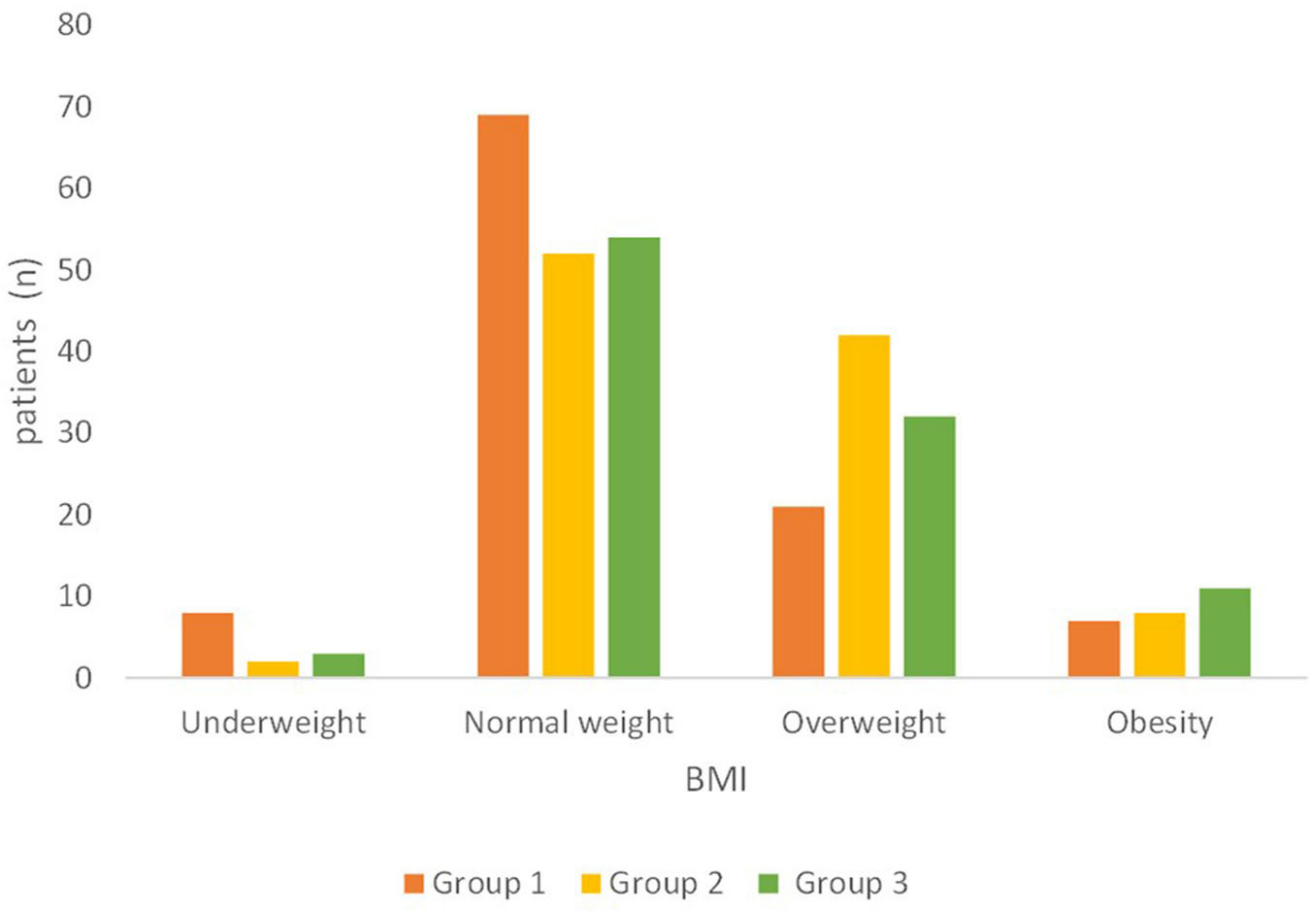
Body Mass Index. Group 1 private practice (*n* = 105, since four patients did not record their height and/or weight); Group 2 healthcare system within the hospital (*n* = 104); Group 3 intra-moenia private practice within the hospital (*n* = 100).

Most of the patients showed a BMI within the range of normality (*p* = 0.02; Fig. 1). Group 1 had the higher number of underweight patients (*n* = 8), while Groups 2 and 3 showed the highest number of overweight or obese patients (*n* = 50 and *n* = 43, respectively).

**Fig. 2:**
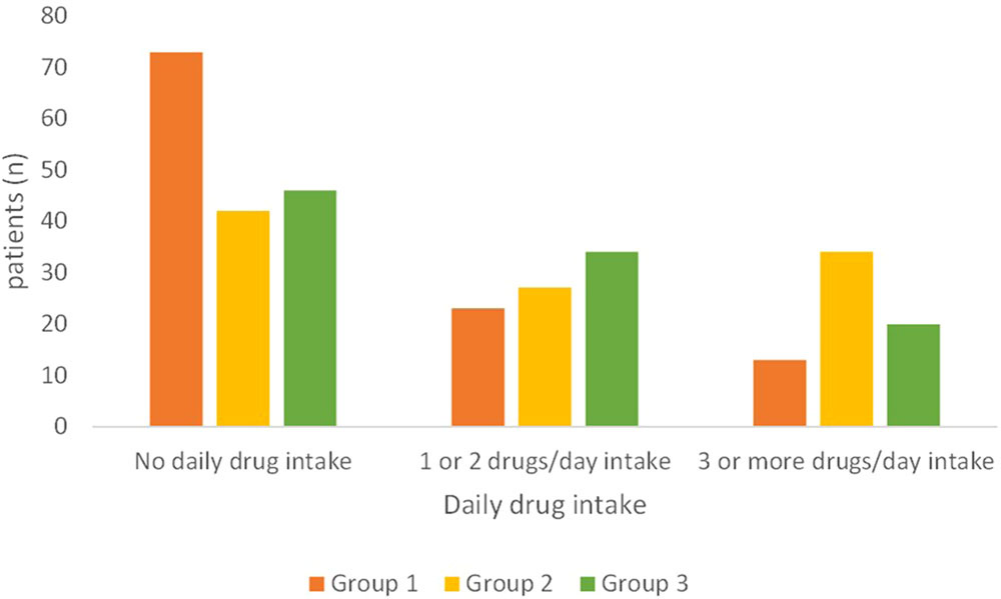
Daily medication intake. Group 1 private practice (*n* = 109); Group 2 healthcare system within the hospital (*n* = 103, one patient did not report any answer); Group 3 *intra-moenia* private practice within the hospital (*n* = 100).

Most of the patients in Group 1 (private practice) reported to not take medications daily (mean= 0.7 ± 1.2 medications/day; range: 0–7 medications) (Fig. 2). In Group 2 (hospital healthcare system) and Group 3 (private practice within the hospital, *intra-moenia*), most patients had an intake of one or more medications daily (mean= 1.8 ± 2.1 medications/day with a range of 0–10 medications and mean = 1.5 ± 2.4 medications/day with a range of 0–10 medications, respectively) (Fig. 2). Group 2 showed a higher number of patients taking 3 or more drugs/day (*p* < 0.01; Fig. 2).

### Answers to the questionnaire

“Have you ever followed a diet?”

This question provides an indication of the previous patient’s propensity and sensibility to receive nutritional counselling. In all the groups, around half of the patients reported to have followed a diet: 63 patients in Group 1, 57 patients in Group 2, and 47 patients in Group 3, without significant differences (*p* = 0.28). In most of cases, the diet was prescribed by a dietician, a nutritionist, or a clinician, while only in a few cases the diet was self-made (Group 1, *n* = 10; Group 2, *n* = 22; Group 3, *n* = 23). In Group 1, the patients who already followed a diet were predominantly females (8 males and 38 females), while, in the other groups, the sexes were balanced.

In Group 1, 15 patients out of 63 (23.8%) who had already received a diet had a BMI out of range: 3 of them were obese (class I *n* = 1; class II *n* = 1; class III *n* = 1) and 11 were overweight, while 1 patient was slightly underweight. Among the patients who reported never having received a diet (*n* = 46), a significantly higher number of patients (*n* = 21; 45.6%; *p* = *0.02*) recorded a BMI out of range compared to the patients who already had a diet: 10 patients were overweight, 4 were *obese* (class I *n* = 2; class II *n* = 2) and 7 were slightly underweight.

In Group 2, 30 patients out of 47 (63.8%) that had already received a diet showed a BMI out of range (three were in class I obesity; 27 patients were overweight). They were significantly more than patients who had not received a diet with a BMI out of range (*n* = 22 out of 57, 38.5%; *p* = 0.02*;* 15 were overweight, five were in class I obesity, and two were slightly underweight).

In Group 3, 29 patients out of 53 (54.7%) that already received a diet had a BMI out of range (twenty were overweight, six were in class I obesity, two in class II obesity, one was slightly underweight), and, among the 47 patients without having received a previous diet, 17 of them (36.1%; *p* = 0.17) had a BMI out of range (twelve were overweight, two were in class I obesity, one was in class II obesity, one was slightly underweight, one was visibly underweight).

“Would you like to receive advice on nutrition, both in general and, specifically, to prevent oral diseases, such as periodontitis, tooth decay and oral cancer?”

**Fig. 3:**
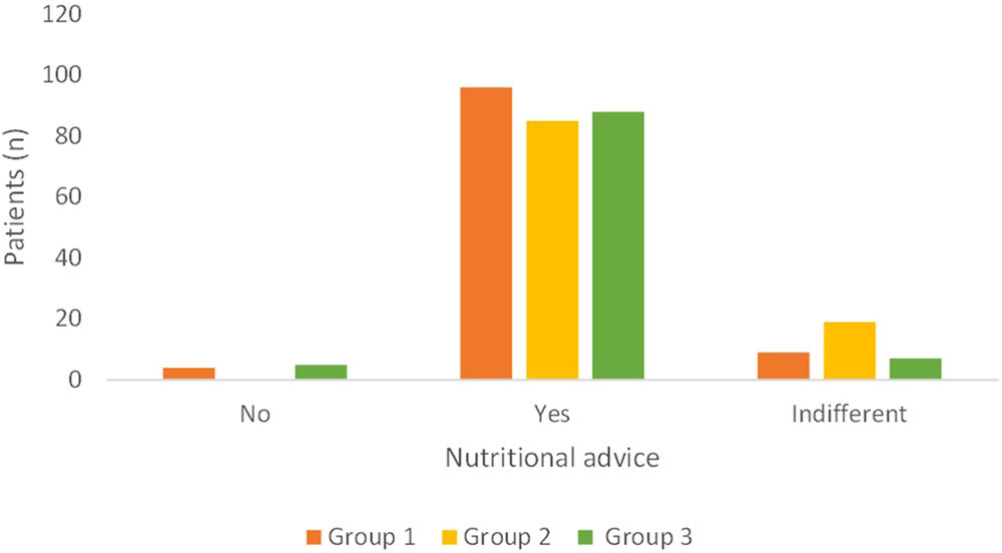
Answers to the question “Would you like to receive advice on nutrition in general, and to prevent oral diseases, such as periodontitis, tooth decay, and oral cancer?”. Group 1 private practice (*n* = 109); Group 2 healthcare system within the hospital (*n* = 104); Group 3 *intra-moenia* private practice within the hospital (*n* = 100).

Most patients (>80%) reported their willingness to receive information regarding nutrition in all the three Groups (Fig. 3; *p* = 0.01). Among the patients who were not interested in having nutritional advice, two were underweight in Group 1, while, in Group 3, one was overweight and one was in class I obesity. Group 2 showed a higher number of patients “indifferent” to receiving the nutritional advice.

“Do you think a dentist, a nutritionist or both should provide such advice to you?”

In Group 1, 5 patients did not reply to this question; thus, only 94 answers were collected.

**Fig. 4:**
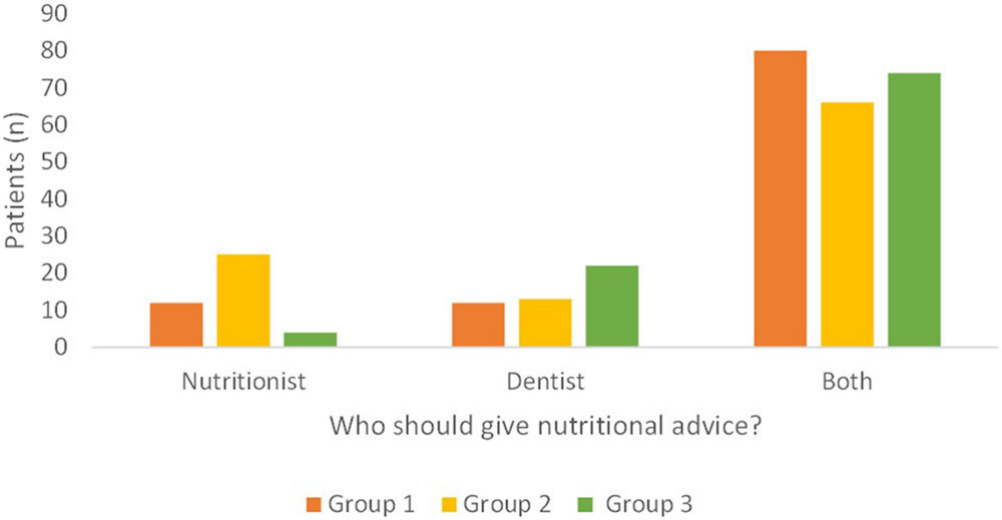
Answers to the question “Do you think a dentist, a nutritionist or both should provide such advice to you?”. Group 1 private practice (*n* = 104, five patients did not report any answer); Group 2 healthcare system within the hospital (*n* = 104); Group 3 *intra-moenia* private practice within the hospital (*n* = 100).

The majority of patients, in all the Groups, identified both the dentist and the nutritionist having the role of providing advice (Fig. 4; *p* < 0.01). In few cases, the role of the dentist alone or the nutritionist alone was recognized, with no difference between the two figures for the Group 1, while the Group 2 recognized the role of the nutritionist more than the Group 3, which reported a predominant importance of the dentist (Fig. 4; *p* = 0.0002).

“Do you think the figure of the nutritionist can be useful in the dental clinics?”

**Fig. 5:**
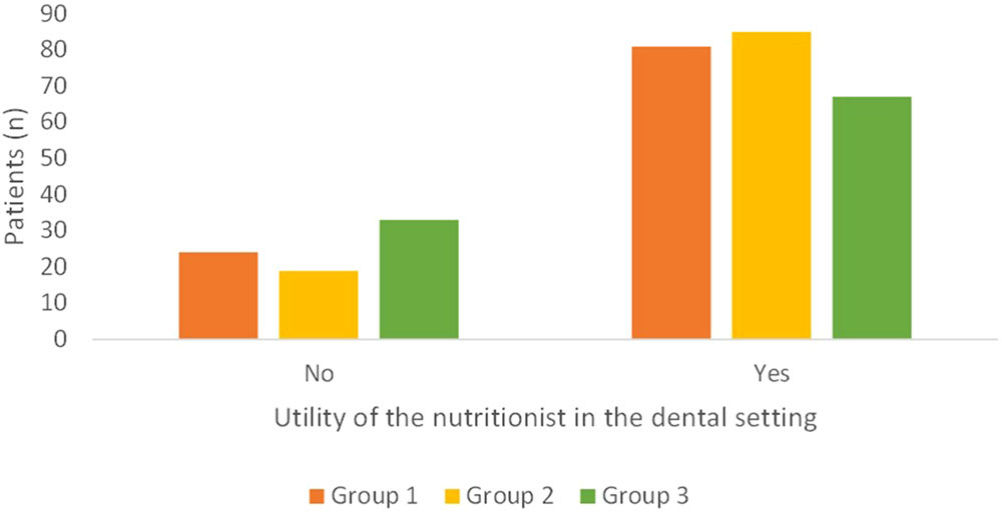
Answers to the question “Do you think the figure of the nutritionist can be useful in the dental clinics?”. Group 1 private practice (*n* = 105; five patients did not reply to this question); Group 2 healthcare system within the hospital (*n* = 104); Group 3 *intra-moenia* private practice within the hospital (*n* = 100).

In all groups, the nutritionist was perceived as useful by most patients in the dental setting, particularly in Group 2, which had patients from the healthcare system (Fig. 5; *p* = 0.05).

“Could you be interested in having, at the dental clinic you are referring to, a nutritionist who can give advice on nutrition in general, also providing a diet useful for managing and preventing diseases other than those of the mouth (systemic diseases, such as diabetes or cardiovascular disorders)?”

**Fig. 6:**
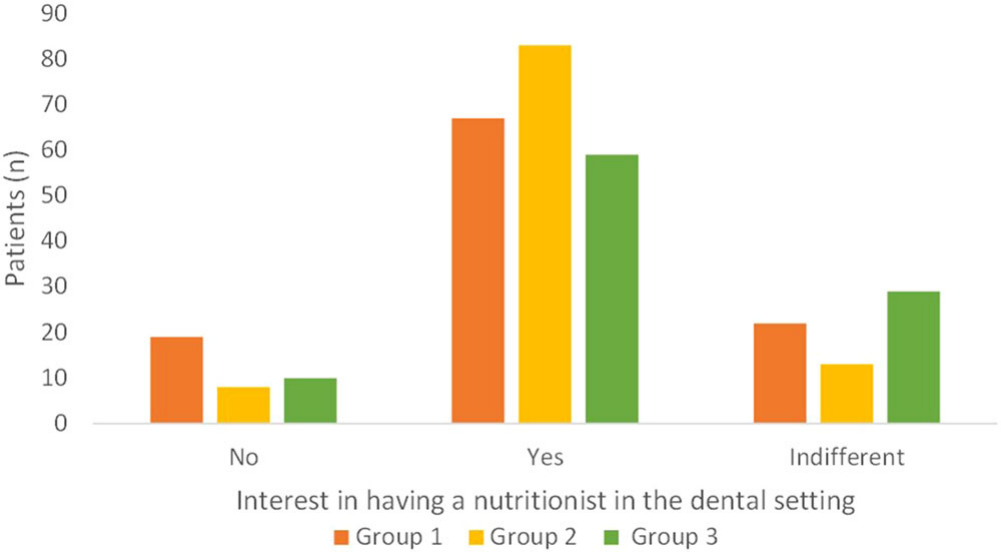
Answer to the question “Could you be interested in having, at the dental clinics you are referring to, a nutritionist who can provide you general advice on nutrition, also elaborating a personalized diet, useful for managing and preventing several systemic non-communicable diseases (such as diabetes or cardiovascular disorders)?”. Group 1 private practice (*n* = 108; one patient did not reply to this question); Group 2 healthcare system within the hospital (*n* = 100; four patients did not provide any answer); Group 3 *intra-moenia* private practice within the hospital (*n* = 100).

Most patients expressed interest in having an expert nutritionist at the dental clinic they refer to (Fig. 6; *p* < 0.01). This was significantly more evident in Group 2, where more than 80% of patients answered positively to this item (Fig. 6; *p* = 0.003). Among the patients who were not interested in having a nutritionist within the dental setting, 8 patients had a BMI out of range in Group 1 (four were overweight; two were in class I obesity; one was in class II obesity; one was slightly underweight); 4 patients were overweight in Group 2; 8 patients in Group 3 were out or range (six were overweight; two were in class I obesity).

“In addition to planning a diet, do you think it would be useful to set a regular follow-up with the nutritionist to monitor the achievements of the dietary goals?”

**Fig. 7:**
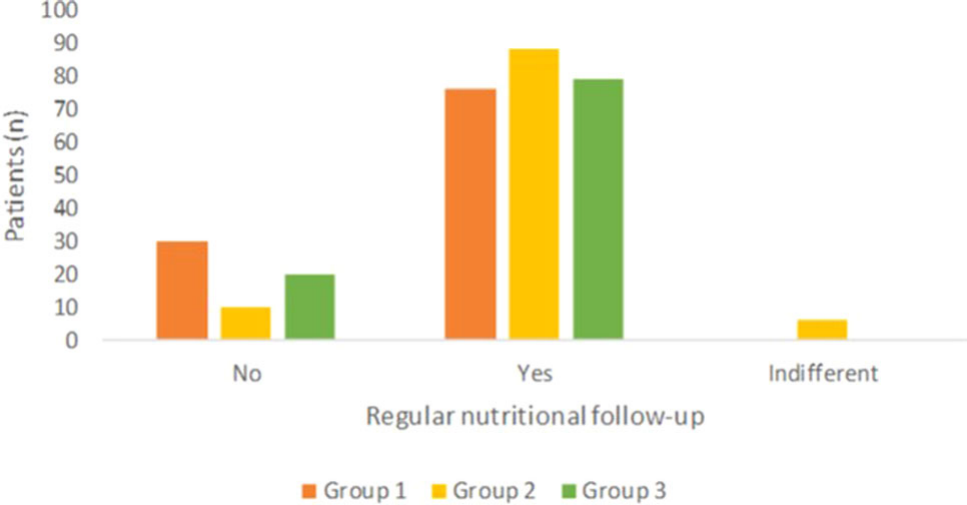
Answer to the question “In addition to planning a diet, do you think it would be useful to set a regular follow-up with the nutritionist to monitor the achievements of the dietary goals?” Group 1 private practice (*n* = 106; three patients did not reply to this question); Group 2 healthcare system within the hospital (*n* = 104); Group 3 *intra moenia* private practice within the hospital (*n* = 99; one patient did not reply to this question).

Planning regular nutritional follow-up was recognized as valuable by most patients, especially in Group 2, where patients responded positively in more than 80% of cases. (Fig. 7; *p* < 0.01).

## Discussion

This survey showed that most dental patients acknowledged receiving nutritional advice from both dentist and nutritionist. They perceived the nutritionist as useful in the dental setting, not only to provide useful information to prevent oral diseases, but also to help preventing systemic diseases, to draw up a diet, and to schedule a periodic follow-up.

The multidirectional link between oral diseases and systemic diseases involves nutrition in a global preventive approach, which considers individual health in a multidisciplinary view [20–22]. Literature supports the importance of dietary habits in the development of caries and periodontitis [19, 23, 24], alongside recent advances in precision nutrition and dietary knowledge to prevent cardiovascular diseases and type 2 diabetes [25–27]. However, most dentists, although recognizing the importance of nutrition in preventing oral diseases, have not received appropriate academic preparation in nutrition counselling [28–30]. Dietary advice is rarely provided by dental practitioners and it can be regarded as a neglected factor for prevention, as influenced by financial considerations, time constraints, and training of dental practitioners [29]. Academic curricula of dental students are often lacking in terms of training in the dietary management of patients [31, 32] and, vice versa, dietetics/nutrition students reported low knowledge in oral sciences [31, 33].

A survey showed that most dentists would consider referring their patients to a dietitian and were open to view the availability of nutrition services as beneficial to their patients [28]. A Cochrane review suggested that one-to-one dietary interventions in dental clinics, to provide nutritional assessment and advice as part of patient management, can change behaviour [34]. Recently, a clinical trial investigated the utility of an inexpensive, educational game for dietary counselling in paediatric patients, emphasizing the importance of including nutritional assessment and advice in the clinical dental routine, even in children [35].

However, no literature is currently available, to the best of our knowledge, on patients’ attitude towards dietary counselling in the dental setting by a nutritionist. In this context, this study is relevant since it explores the propensity of dental patients to receive personalized nutritional information, useful for promoting both oral and systemic health, emphasizing the importance of global wellness and prevention.

Within the limitations of this cross-sectional study, which included a population sample from a single Italian region (Lombardy) collecting self-reported data using a pilot questionnaire, the dental patient perceived the presence of a nutritionist in the dental setting positively, in order to receive dietary advice and information at the dental clinics. In few cases, the patients reported no utility in receiving nutritional support. The introduction of the nutritionist in the dental clinics and a general higher attention of the dentist to diet and nutrition could help intercepting early pictures of metabolic syndrome as well as preventing its onset, thus reinforcing the role of the dental setting in the oral and systemic health promotion. The collection of BMI values related to each patient, in particular, represents a first screening measure to identify overweight and obesity in the dental setting, since these conditions can be related to dental diseases, such as periodontitis, besides systemic non-communicable diseases [9, 11–13].

The three groups of patients, involved in the study, included the adult individuals that usually refers to the dental clinics seeking for care, both in public and private dental settings. The analysis of the three different dental settings is relevant, since patients, referring to each of them, have, on average, different socio-economic backgrounds and putatively different dietary patterns, therefore a supposedly different sensitivity and propensity to receive nutritional advice. The two provinces of participants’ provenience, Milan and Lecco, embody, respectively, a large, highly urbanized metropolitan area and a smaller urban reality. Although the convenience sampling here applied and the limited geographical distribution (only provinces of the North of Italy were involved) hinder the direct generalizability of results to the general population, the study shows the overall trend of interest towards this topic among dental patients. Further multicentric studies will be pivotal in corroborating or not these results in different topographical areas with different socio-cultural entities. Future research should also focus on providing specific validated questionnaires on the topic.

## Conclusions

Dental patients perceived the dental setting as adequate to receive nutritional advice from both nutritionists and the dental staff. They also reported the willingness to obtain information and to follow nutritional programs not only for preventing oral diseases, but also for preventing systemic diseases.

Considering the increasing importance of nutrition in the prevention of oral and systemic diseases, a multidisciplinary approach is pivotal, where the dental clinics could play a role in periodical screening and check-up, and where the nutritionist joins the dental team and becomes part of the management of the dental patient.

### Supplementary information

Supplementary Appendix 1

## Data Availability

Data is available upon request due to privacy or ethical restrictions.
