# Supplementary material for: Survey on patients’ attitude towards the nutritional counselling in the dental setting
Source: BDJ Open. 2024 Jun 11;10:47. doi: 10.1038/s41405-024-00229-0 (PMC11166955; doi:10.1038/s41405-024-00229-0)
Supplement: Supplementary file 1 — Supplementary Appendix 1 [file 41405_2024_229_MOESM1_ESM.pdf]

Supplementary Appendix 1. Questionnaire self-administered to patients referring to dental clinics about their attitude towards receiving a nutritional support in dental setting.

Age	
Sex	
Height	
Weight	
Job	
Number of medications assumed daily	

---

1. Have you ever followed a diet?
   - Yes
   - No

If yes, please specify the reason:

- prescribed by a doctor (general practitioner..... or specialist in ....)
- prescribed by a dietician medical doctor
- prescribed by a dietician / nutritionist
- “do it yourself” diet

2. Would you like to receive advice on nutrition in general, and to prevent oral diseases, such as periodontitis, tooth decay and oral cancer?
   - Yes
   - No
   - Indifferent

---

3. Do you think a dentist, a nutritionist or both should provide such advice to you?
   - Only dentist
   - Only nutritionist
   - Both
4. Do you think the figure of the nutritionist can be useful in a dental clinic?
   - Yes
   - No
5. Could you be interested in having, at the dental clinic you are referring to, a nutritionist who can give advice on nutrition in general, also providing a diet useful for managing and preventing diseases other than those of the mouth (systemic diseases, such as diabetes or cardiovascular disorders)?
   - Yes
   - No
   - Indifferent
6. In addition to planning a diet, do you think it would be useful to set a regular follow-up with the nutritionist to monitor the achievements of the dietary goals?
   - Yes
   - No
